# ^1^H NMR Spectroscopy and MVA Analysis of *Diplodus sargus* Eating the Exotic Pest *Caulerpa cylindracea*

**DOI:** 10.3390/md13063550

**Published:** 2015-06-05

**Authors:** Sandra A. De Pascali, Laura Del Coco, Serena Felline, Ernesto Mollo, Antonio Terlizzi, Francesco P. Fanizzi

**Affiliations:** 1 Department of Biological and Environmental Science and Technologies (Di.S.Te.B.A.), University of Salento, Prov.le Lecce-Monteroni, 73100 Lecce, Italy; E-Mails: sandra.depascali@unisalento.it (S.A.D.P.); laura.delcoco@unisalento.it (L.D.C.); serena.felline@unisalento.it (S.F.); antonio.terlizzi@unisalento.it (A.T.); 2 Institute of Biomolecular Chemistry, CNR, Via Campi Flegrei 34, 80078 Pozzuoli, Naples, Italy; E-Mail: emollo@icb.cnr.it

**Keywords:** *Caulerpa cylindracea*, *Diplodus sargus*, fish plasma, metabolic status, NMR spectroscopy, multivariate analysis, PCA, OPLS, OPLS-DA

## Abstract

The green alga *Caulerpa cylindracea* is a non-autochthonous and invasive species that is severely affecting the native communities in the Mediterranean Sea. Recent researches show that the native edible fish *Diplodus sargus* actively feeds on this alga and cellular and physiological alterations have been related to the novel alimentary habits. The complex effects of such a trophic exposure to the invasive pest are still poorly understood. Here we report on the metabolic profiles of plasma from *D. sargus* individuals exposed to *C. cylindracea* along the southern Italian coast, using ^1^H NMR spectroscopy and multivariate analysis (Principal Component Analysis, PCA, Orthogonal Partial Least Square, PLS, and Orthogonal Partial Least Square Discriminant Analysis, OPLS-DA). Fish were sampled in two seasonal periods from three different locations, each characterized by a different degree of algal abundance. The levels of the algal bisindole alkaloid caulerpin, which is accumulated in the fish tissues, was used as an indicator of the trophic exposure to the seaweed and related to the plasma metabolic profiles. The profiles appeared clearly influenced by the sampling period beside the content of caulerpin, while the analyses also supported a moderate alteration of lipid and choline metabolism related to the *Caulerpa*-based diet.

## 1. Introduction

It is well known that non autochthonous species can cause severe changes in marine ecosystem such as altering availability or quality of nutrients and other resources, changing habitat structure and affecting genetic flow or reproductive performance [1,2,3]. Among the copious foreign species reported in the Mediterranean basin [4], great attention was paid to the green algae *Caulerpa cylindracea* Sonder, previously identified as *Caulerpa racemosa* var. *cylindracea* (Sonder), due to the remarkable change induced in the invaded area. In the last decades, the invasion of the non-native *C. cylindracea* was increasingly at the center of hot discussions for both scientific and general public communities, because of its rapid and broad spread, which is inducing profound changes in the bottom of the sea landscape [5]. Native to the southwestern coast of Australia, *C. cylindracea* is present in most parts of the Mediterranean Sea, where it has also invaded many Marine Protected Areas (MPAs) [6,7]. *C. cylindracea* can modify habitat structure and associated fauna, altering food webs and trophic guilds with strong negative effects on native benthic communities. Recently, the analysis of fish gut contents carried out in several areas invaded by *C. cylindracea*, has showed that the invasive algae has been included into the diet of the white seabream *Diplodus sargus*, becoming its most abundant food resource [8,9,10]. To date, and for reasons that still need to be fully elucidated, *Spondyliosoma cantharus* and *Diplodus sargus* are the only Mediterranean sparid fish species whose feeding activity on the invasive algae has been measured and documented. The switch from a diet based on a variety of animal and plant sources to one mostly centered on the invasive alga, has been shown to influence organoleptic properties and nutrition quality of this fish resource, which is relevant for the market, with a reduction of polyunsaturated fatty acids of the *n*-3 and *n*-6 series in muscles of white seabream due to the *C. cylindracea*-based diet [9]. More specifically, an impoverishment of the essential and beneficial fatty acids for human nutrition, such as the docosahexaenoic (C22:6), eicosapentaenoic (C20:5) and arachidonic (C20:4) acid, was observed in *D. sargus* eating *C. cylindracea* [9]. Noteworthy, the nutritional value, taste and flavor of the fish fillets strongly depend on their fat content, fatty acid composition and muscle amino acids, which are all clearly influenced by the animal dietary history [10]. Furthermore, *C. cylindracea* contains bioactive metabolites possibly affecting the health of the fish [11], such as the sesquiterpene caulerpenyne, which has been suggested to act both as chemical deterrent against herbivores and allelochemical in interspecific competition [12], and the red bisindolic pigment caulerpin that, however, did not seem produce acute toxicity [13]. The production of secondary metabolites by the alga changes in line with the seasons: the highest level was found in summer and autumn whereas the lowest in winter, in agreement with the vegetative activity of the algae [14]. The new trophic relationship between *C. cylindracea* and *D. sargus*, has also been confirmed on a chemical basis by observing the accumulation of caulerpin in the fish [15]. The presence of caulerpin in fish tissues has been considered evidence of *C. cylindracea* consumption and then used as a marker of trophic exposure to the alien seaweed. Furthermore, the caulerpin levels have been correlated to some cellular and physiological alterations which included: (i) slight modulation of antioxidant defenses (glutathione reductase and glutathione levels); (ii) increased enzymatic activities of cytochrome P450, acetylcholinesterase and acyl CoA oxidase activities; (iii) changes of hepatosomatic and gonadosomatic indices [8,16]. However, the possible causal relationship between the different algal metabolites and the above observed alterations have not yet been fully clarified, and will require cross-disciplinary investigations ranging from natural product chemistry to chemical ecology, from pharmacology to eco-toxicology. Meanwhile, it becomes increasingly urgent to create analysis tools that enable rapid measurement of the metabolic state of the fish exposed to seaweed, which can facilitate the monitoring of the effects on its new alimentary habits. A number of studies have used ^1^H NMR based metabolomics to identify metabolic differences, caused by a range of inherent and external factors, which could influence the metabolic status of marine organisms (fish) [17,18,19,20]. NMR (Nuclear Magnetic Resonance) and Mass Spectrometry (MS) based metabolomic of biofluids and/or cell extracts has the enviable property of providing a global profiling tool for monitoring disease status or time-related metabolic phenomena, by comparison with healthy organisms biochemical profiles. The usefulness of metabolomics for the evaluation of xenobiotic toxicity effects has recently been comprehensively explored [21]. Nevertheless, the limited number of studies conducted to date has shown the general approach of metabolic profiling to be highly discriminatory also in assessing fish physiology. ^1^H NMR spectroscopy and multivariate analysis of blood plasma have been applied in order to evaluate pathogenic status of Atlantic salmon (*Salmo salar*) [17,18]. This approach led us to search for any correlation between changes in the metabolic profiles of *D. sargus* and its trophic exposure to *C. racemosa* evidenced by the accumulation of caulerpin in the fish tissues exposed to *C. cylindracea*. Therefore, caulerpin accumulation levels in fish liver, measured in our previous work focusing on biomarker analyses [8,16], have been here, instead, related to changes in the metabolic profiles of the plasma collected from the same fish sample using ^1^H NMR spectroscopy and multivariate analysis both unsupervised (Principal Component Analysis, PCA) and supervised (Orthogonal Partial Least Square, PLS, and Orthogonal Partial Least Square Discriminant Analysis, OPLS-DA). Given that the plasma samples were collected in two periods, June and October 2012, along three areas of the Southern Apulian coast (SE, Italy), Brindisi (BR), Porto Cesareo (PC) and Torre Guaceto (TG), differently characterized in terms of *C. cylindracea* abundance, the metabolic profiles were also analyzed to evaluate possible variations related to the seasonal fluctuations of *C. cylindracea.*

## 2. Results and Discussion

### 2.1. NMR Spectroscopy and MVA (Multivariate Analysis)

**Figure 1:**
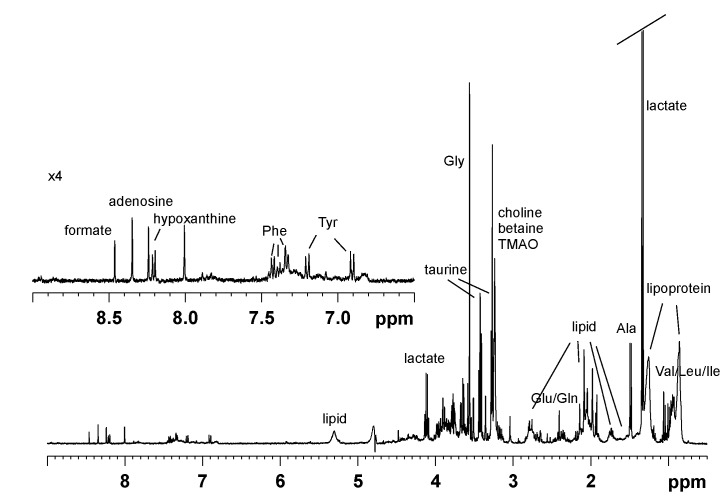
Representative ^1^H NMR CPMG spin-echo spectrum of fish blood plasma in D_2_O.

In order to understand the alga impact on seabream plasma metabolomic profiles, 41 blood samples of *D. sargus* fished in two periods (June and October) in invaded areas of Apulia (Brindisi, BR, Porto Cesareo, PC, Torre Guaceto, TG) were collected. The fish exposure to the invasive alga was previously assessed by measuring the caulerpin level in the animal liver [16], as summarized in Table 1, while the plasma samples were classified according to caulerpin content in the liver of the corresponding fish individuals. Not considering the sampling site, the samples were thus divided in four groups corresponding to caulerpin fish content (see Experimental Section). A typical one-dimensional (1D) ^1^H NMR spectrum of the fish blood plasma (selected for graphical purpose due to the presence of most of the metabolite NMR signals) is reported in Figure 1. More than twenty metabolites were identified in the ^1^H NMR spectra and assigned on the basis of 2D NMR spectra analysis (2D ^1^H *J*res, ^1^H COSY, ^1^H ^13^C HSQC and HMBC) and by comparison with published data [17,18,19,20,21,22]. The presaturated ^1^H NMR CPMG spectrum is dominated by amino acids, organic acid and broad resonances originating mainly from lipoprotein lipids. Despite the use of CPMG sequence [23], some broad resonances from lipids in lipoproteins can be observed in the blood plasma NMR spectra. This is due to the flexibility of certain parts of such macromolecules making the T2 for those specific protons longer if compared with the rest of the molecule [21]. Although lipoproteins in blood serum or plasma have been extensively studied by using NMR spectra deconvolution techniques [24] and their analysis could be interesting for further studies, we focused our attention on small metabolites plasma profiles. Relevant ^1^H NMR data are reported in Table 2. Metabolic profiles of plasma characterized by ^1^H NMR spectroscopy were therefore studied with multivariate analyses (PCA, OPLS and OPLS-DA).

**Table 1:** Concentration of caulerpin in liver of white seabrem *D. sargu*s sampled from Torre Guaceto (TG), Brindisi (BR) and Porto Cesareo (PC), in June (a) and October (b) 2012. Values of caulerpin concentrations are expressed in μg/g of dry weight (μg/g).

Caulerpin (μg/g)				Caulerpin (μg/g)				Caulerpin (μg/g)			
June		October		June		October		June		October	
PC1a	3.72	PC1b	0.00	BR1a	7.41	BR1b	29.09	TG1a	0.00	TG1b	10.07
PC2a	3.98	PC2b	0.00	BR2a	8.78	BR2b	3.2	TG2a	0.00	TG2b	0.00
PC3a	5.09	PC3b	0.00	BR3a	7.07	BR3b	8.8	TG3a	0.00	TG3b	27.94
PC4a	31.43	PC4b	0.00	BR4a	54.58	BR4b	28.93	TG4a	4.6	TG4b	78.33
PC5a	29.07	PC5b	0.00	BR5a	43.82	BR5b	54.6	TG5a	6.72	TG5b	39.02
	-	PC6b	0.00	BR6a	30.8	BR6b	5.5		-	TG6b	40.98
	-	PC7b	0.00		-	BR7b	114.42		-	TG7b	93.6
	-	PC8b	6.34		-	BR8b	235.75		-	TG8b	114.08
	-	PC9b	55.02								
*mean*	14.66		6.82		25.41		60.04		2.26		50.5
*sem*	6.38		5.72		8.48		28.23		1.43		14.39

**Table 2:** Chemical shift (δ) and assignment of metabolite resonances in the ^1^H NMR spectrum of fish blood plasma.

Metabolites	δ (ppm)
cholesterol	0.74 (m ^a^, CH_3_)
lipoprotein	0.83 (t), 0.85 (t), 0.86 (t), 0.88 (t), 0.93 (t), 0.98 (t), 1.05 (t) (C*H*_3_CH_n_)
leucine	0.96 (d, CH_3_), 0.98 (d, CH_3_), 1.72 (m, CH), 1.73 (m, CH)
isoleucine	0.94 (t, CH_3_), 1.02 (d, CH_3_)
valine	1.00 (d, CH_3_), 1.05 (d, CH_3_)
lactate	1.33 (d, CH_3_), 4.11, (q, CH)
alanine	1.48 (d, CH_3_), 3.80 (m, CH)
lipoprotein	1.20 (m), 1.25 (m), 1.26 (m), 1.31 (m) (C*H*_2_)_n_
acetate	1.94 (s, CH_3_)
lipid	1.51 (m), 1.55 (m) (C*H*_2_CH_2_CO)
lipid	1.44, 1.52, 1.68 (m) (C*H*_2_CH_2_C=C)
lipid	1.98, 2.04, 2.08, 2.14, 2.19 (C*H*_2_C=C)
lipid	2.23 (m), 2.35, 2.42, 2.46, (C*H*_2_CO)
lipid	2.65, 2.69, 2.76, 2.77, 2.78 (m) (C=CC*H*_2_C=C)
lipid	5.31 (m) (*H*C=C*H*)
methionine	2.14 (s, S-CH_3_), 2.66 (t, CH_2_), 3.80 (t, CH)
glutamate	2.07 (m, CH_2_), 2.35 (m, CH_2_)
glutamine	2.14 (m, CH_2_), 2.45 (m, CH_2_)
citrate	2.54 (d, CH_2_), 2.67 (d, CH_2_)
choline	3.24 (s, N(CH_3_)_3_)
TMAO	3.27 (s, N(CH_3_)_3_)
betaine	3.27 (s, N(CH_3_)_3_), 3.91 (s, CH_2_)
taurine	3.27 (t, CH_2_NH), 3.42 (t, CH_2_SO_3_)
glycine	3.59 (s, CH)
glycerol	3.56 (dd, CH_2_), 3.66 (dd, CH_2_), 3.89 (m, CH)
α-glucose	5.24 (d, CH)
β-glucose	4.65 (d, CH)
tyrosine	6.91 (m, C3,5H ring), 7.20 (m, C2,6H ring)
phenylalanine	7.43 (m, C3,5H), 7.38 (m, C4H), 7.33 (m, C2,6H)
hypoxanthine	8.20 (s, C2H), 8.21 (s, C8H)
adenosine	8.24 (s, C2H), 8.35 (s, C8H)
formate	8.46 (s, CH)

^a^ Letters in parentheses indicate the peak multiplicities; s, singlet; d, doublet; t, triplet; dd, doublet of doublet; m, multiplet.

**Figure 2:**
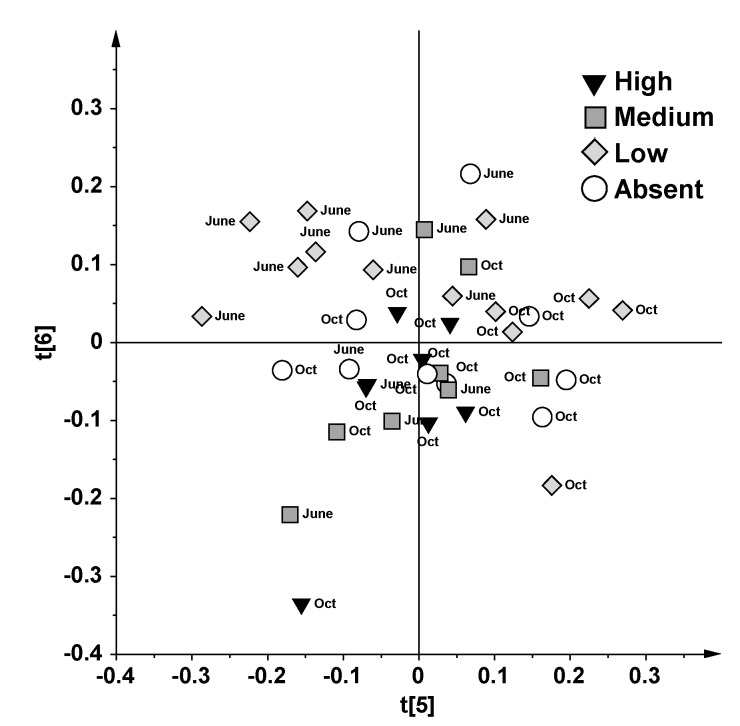
t5/t6 PCA score plot obtained from ^1^H NMR CPMG data for 39 plasma samples. Relating to the caulerpin liver content, the samples were divided in Absent (0 μg/g d.w.), Low (< 10 μg/g d.w.), Medium (>10, <50 μg/g d.w.) and High (>50 μg/g d.w.).

PCA allowed us to obtain a general overview of the natural data grouping and to show similarities and differences in biochemical profiles. The original dataset (185 buckets from the spectral region 9.50–0.50 ppm) was rearranged in a new multivariate coordinate space in which the dimensions are ordered by decreasing explained variance of the considered data. The principal components were displayed as a set of scores (t) and a set of loadings (p), which highlight clustering or outliers and influence of input variables on t, respectively. A diagnostic showing strong outliers is given by Hotelling’s T2 [25]. This statistic is a multivariate generalization of the Student’s *t*-test, and provides a check for observations adhering to multivariate normality. In the Hotelling’s T2 plot (Supplementary Figure S1) two samples were discarded and excluded from further considerations due to their marked distance from the model. However, no relevant trends among samples were observed in the PCA models, except for a tendency to separate by seasonality observed in the t5/t6 score plot (Figure 2) and related loading plot (Supplementary Figure S2).

In order to improve the separation among samples based on maximizing covariance between the measured data (*X*) and the response variable (*Y*), OPLS-DA models were also studied. By this method, the identity of each group of samples is specified in the model such that the maximum variance of the groups can be attained in the hyperspace.

**Figure 3:**
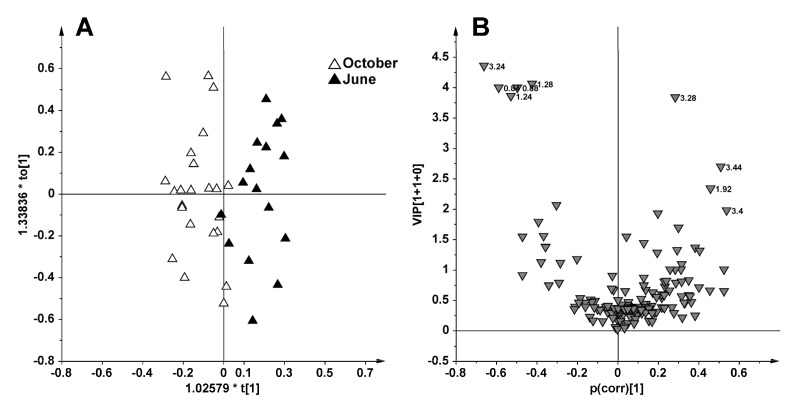
OPLS-DA (**A**) and p(corr)/VIP (**B**) score plot obtained from ^1^H NMR CPMG data for 39 plasma samples using as responsible variable (Y) the fishing period (June and October).

The model evaluation parameters and the extraction methods of metabolic results are fully described in the experimental section. Two performance indicators were used to assess the supervised model complexity and overfit degree: The cross validation (CV) and the response permutation test (*n* = 400). An informative two components (1 predictive and 1 orthogonal) model was obtained with OPLS-DA applied to all 39 samples using the fishing period (June and October) as a responsible variable (Y) (model diagnostic parameters: *R*^2^Xcum = 0.39, *R*^2^Ycum = 0.72, *Q*^2^cum = 0.47 and and *p*[CV-ANOVA] = 1.8 × 10^−4^) (Figure 3A). In order to identify the subset of NMR buckets (and related metabolites NMR signals) with the highest influence in the model, variable selection using a combination of VIP (Variable Importance for the Projection) (VIP > 1.0) and p(corr) (|p(corr)| > 0.5) was performed. The p(corr)/VIP score plot (Figure 3B) shows that the separation between the two fishing periods was ascribable to a higher content of choline (bucket at 3.24 ppm) and lipids (buckets at 0.84, 0.88, 1.2, 1.28 ppm) and a lower content of taurine (buckets at 3.28, 3.4, 3.44 ppm) and acetate (1.92 ppm) for the samples obtained from October with respect to those from June captures.

**Figure 4:**
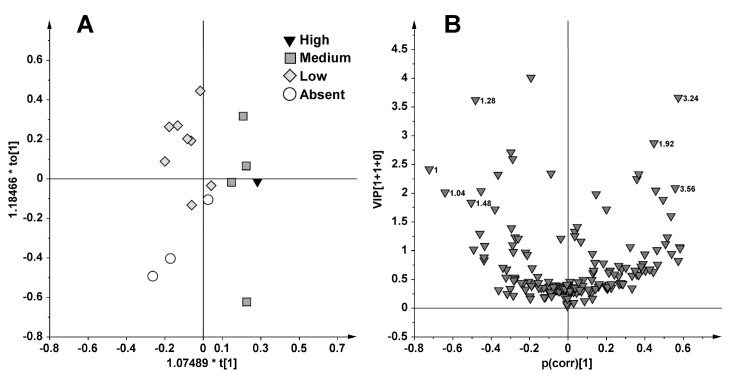
OPLS (**A**) and p(corr)/VIP (**B**) score plot for samples fished in June using as responsible variable (Y) the liver caulerpin content (Absent = 0 μg/g d.w., Low < 10 μg/g d.w., Medium >10, <50 μg/g d.w. and High >50 μg/g d.w.).

**Figure 5:**
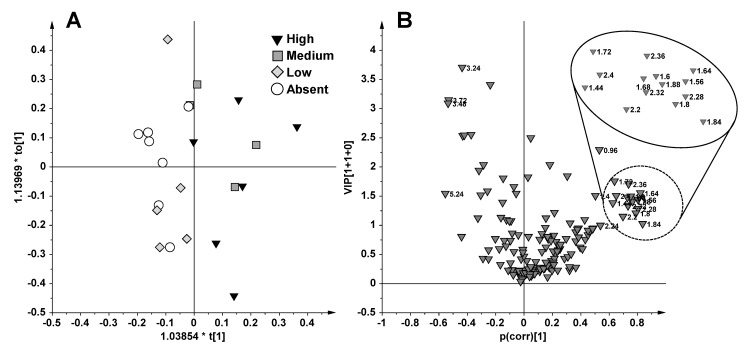
OPLS (**A**) and p(corr)/VIP score plot (**B**) for samples fished in October using as responsible variable (Y) the liver caulerpin content (Absent = 0 μg/g d.w., Low < 10 μg/g d.w., Medium >10, <50 μg/g d.w. and High >50 μg/g d.w.).

In this regard, the samples from June fishing (16) were further analyzed separately from those collected in October (23) by OPLS using as X matrix 185 buckets of ^1^H NMR spectra (columns) for each row (16 and 23 for June and October, respectively) and as responsible variable (Y) the liver content of caulerpin. The OPLS score plot (Figure 4A) of the samples fished in June gave a model (1 predictive and 1 orthogonal components) with a *R*^2^Xcum = 0.42, *R*^2^Ycum = 0.78, *Q*^2^cum = 0.34 and *p*[CV-ANOVA] = 0.29. Looking at the p(corr)/VIP score plot (Figure 4B) the High and Medium separate from the Low and Absent samples due to higher signals at 3.24, 3.56 and 1.92 ppm, assigned to choline, glycine and acetate, respectively. A higher content of valine and alanine (1.00, 1.04 and 1.48 ppm) was observed in sample classified as Absent and Low. In the OPLS analysis of the samples collected in October, the sample with the highest content of caulerpin located at the far values extremity as a single observation (235.75 μg/g d.w.) was excluded for achieving approximately normally distributed data. An OPLS model was then obtained (Figure 5A) for the samples fished in October using as responsible variable (Y) the caulerpin liver content (*R*^2^Xcum = 0.24, *R*^2^Ycum = 0.79, *Q*^2^cum = 0.37 and *p*[CV-ANOVA] = 0.077). Analysis of the p(corr)/VIP score plot (Figure 5B) showed higher content of choline (bucket at 3.24 ppm) and α/β glucose (buckets at 5.24, 3.88, 3.84, 3.76, 3.72, 3.48 ppm) for the Absent and Low whereas the High and Medium samples exhibited higher signals in the range 2.40–1.80 ppm and 1.72–1.56 ppm, attributed to increased lipids concentration. However, both June and October samples give OPLS models with good descriptive but rather weak predictive ability.

### 2.2. Discussion

In the last years invasions of non-native species have been identified as the major threat for biodiversity and ecosystem functioning [26,27]. The green alga *C. cylindracea* was introduced in the Mediterranean Sea from the southwestern coast of Australia, and it has rapidly invaded benthic communities with severe negative effects on native assemblages [28]. Probably the production of secondary metabolites by *C. cylindracea* is the key for its advantageous interactions with competing native macrophytes, and as anti-predatory defence against grazers, but there is still a limited knowledge on the ecological role of the produced molecules. It is noteworthy, however, that the most studied secondary metabolites from *C. cylindracea*, namely the above mentioned compounds caulerpenyne and caulerpin, have already shown a panel of critical biological activities [11].

*C. cylindracea* has been recently observed to frequently occur in the stomach content of the white sea bream *D. sargus*, which typically feed on shellfish and other benthic invertebrates, while a significant accumulation of caulerpin in the fish tissues has been also detected [8,15]. The level of caulerpin in *D. sargus* has been thus used as a trophic marker and related to the alteration of some enzymatic pathways (including antioxidants, cytocrome P450 and acyl CoA oxidase activities). In this regard, a potential role of algal metabolites was hypothesized in modulating possible adverse effects on the health of *D. sargus* [8]. Evaluating the individual and combined effects of the metabolites contained by *C. cylindracea* will certainly require the interdisciplinary efforts of researchers working at the intersection of biology and chemistry. However, rapid tools are also needed to measure the set of metabolic alterations occurring in the fish for the on time development of appropriate preservative management measures. Actually, the metabolomics based approach that characterizes the present work stems from this needs.

Despite the fact that the NMR spectroscopy has been widely applied to metabolomic studies of humane biological fluids and widely to agricultural matrices [29], to the best of our knowledge, only a few studies on fish blood plasma by NMR spectroscopy and multivariate data analysis have been reported [17,18,19,20].

In this work, for the first time, the metabolic profiles of *D. sargus* plasma were studied by ^1^H NMR spectroscopy and MVA in relation to the invasion of exotic alga *C. cylindracea*. Interestingly, as already reported by Gorbi *et al.* [16], the studied samples were mainly grouped according to the sampling period, thus excluding severe damage to the health condition of white seabreams due to accumulating caulerpin. On the other hand, the results here reported showed that the *D. sargus* captured in October (those with the highest liver caulerpin value), exhibited higher choline and lipoproteins and lower taurine and acetate plasma levels with respect to the fishes sampled in June.

Choline is a precursor of several membrane phospholipids (e.g., phosphatidylcholine and sphingomyelin), the methyl donor betaine, and the neurotransmitter acetylcholine. Recently, Karakach *et al.* [18] reported an increment of choline concentration in *Salmo salar* plasma due to long term handling stress. This change could be the result of an overall increase in plasma membrane turnover and/or phosphatidylcholine (PC) breakdown. Moreover, variations in lipoprotein and lipid contents have been also reported during stress [30]. Probably, the increased or decreased lipid metabolism could be due to an animal attempt to deal with and adapt to the stress, since lipids and fatty acids are the preferred sources of energy for growth and reproduction [19].

Taurine is a free amino acid that is present in large amounts in various marine fish tissues, however it is not considered an essential amino acid because it can be synthesized by fish [31]. In mammals, taurine plays important roles in osmoregulation, bile acid conjugation, membrane stabilization, hormone release, neurotransmitter modulation, and as an antioxidant [32]. For marine species, taurine plays a key role in osmoregulation and typically account for more than 50% of the free amino acid pool [33]. As observed in mammals, it has been also reported that the rainbow trout synthesizes taurine from cysteine through mainly the l-cysteinesulphinate decarboxylase enzymatic activity [34]. Activity of this enzyme varies in fish depending upon species and size. The wild carnivorous fishes, such as *D. sargus*, are able to assume relatively large quantities of taurine as they eat abundant animal tissues, but this does not occur when diets contain large amounts of plant protein sources, which are naturally poor in taurine. Fish grown in sea water may undergo a greater demand for dietary taurine than fish held in fresh water and a fish’s ability to convert cysteine to taurine may be based on the environmental salinity conditions [35]. Moreover, a significant impact of taurine supplementation on growth and feed efficiency of juvenile cobia sea fish (*Rachycentron canadum*) has been reported [36].

As a further level of investigation, OPLS analyses were performed considering separately the sampling period (June and October). Four bioaccumulation groups were also defined in this further study (Absent, 0 μg/g d.w.; Low, <10 μg/g d.w.; Medium, 10–50 μg/g d.w.; High >50 μg/g d.w.) to evaluate differences of biomarker responses in each sampling period according to the methodology used in previous works [8,16]. Despite both June and October OPLS models not showing good predictive ability, significant differences for some metabolites content was observed. The High and Medium samples showed the highest concentration of choline, glycine and acetate in June and the highest content of lipids in October; whereas the Absent and Low specimens resulted with high alanine and valine in June and the highest level of choline and glucose in October.

Glycine together with serine take part in gluconeogenesis, sulfur amino acids metabolism, one-carbon unit metabolism, fat digestion [37], and also stimulate food intake in many fishes [36]. Moreover, glycine may regulate gene expression in fish, thus improving the efficiency of nutrient absorption and anabolic processes. Interestingly, glycine has a critical role in the osmoregulatory responses of fishes and shellfishes to environmental stress [38,39].

Alanine and valine together with lactate and other aminoacids are important precursors of glucose formation through gluconeogenis [40]. Then, a decrease of these metabolites could suggest that the gluconeogenesis was stimulated. Plasma concentrations of glucose are regulated by complex interactions of hormones such as glucagon and cortisol. An increase of blood glucose has been reported as general stress response indicator in fish [19]. Moreover, a hypoglycemic condition caused by a decrease of plasma glucose concentrations has been described in a study where the effect of sublethal concentrations of monocrotophos on *Channa punctatus* (Bloch) was investigated [41].

It is worth noting that most of the works related to fish metabolite analyses consider systems where the influencing factors are usually under strict control (specific diet, handling conditions, sewage exposition, *etc.*) [18,19,20]. The distinctive characteristic of the present work is mainly related to the evaluation of the variation of the metabolic profiles of a fish as an effect of its alimentary behavior in the field, which has been monitored by using the secondary metabolite caulerpin as a trophic marker indicating fish exposition to the invasive alga. This has allowed the detection, on the whole, of metabolic abnormalities in the plasma of *D. sargus* associated with a diet based on the green alga *C. cylindracea*.

## 3. Experimental Section

### 3.1. Sample Collection

The invasive *C. cylindracea* shows a seasonal cycle, reaching the maximum development from summer to autumn followed by a drastic regression in winter [42]. Since the seasonal variations in the growth rate were highly significant, two sampling periods, June and October, were considered for *D. sargus* plasma sampling, namely June and October 2012. Sampling locations included the coast between Brindisi and Lecce (BR), and the Marine Protected Areas (MPAs) of Porto Cesareo (PC) and Torre Guaceto (TG), which are characterized by a very similar compositional structure of the rocky sessile assemblages [43,44]. About the colonization of the seabed, *C. cylindracea* dominates the benthic community of TG and BR, whereas it is less abundant in PC. We were able to collect blood samples from the caudal vein of 41 specimens of *D. sargu*s (16 fished in June: 6 from BR, 5 from PC, 5 from TG; 25 fished in October: 8 from BR, 9 from PC, 8 from TG) stored in a polystyrene box containing ice until transportation to the laboratory. Blood samples were then centrifuged at 4 °C, 3000× *g* for 10 min. After centrifugation, the plasma was collected and lyophilized for the NMR metabolomics profiling. Each lyophilized sample was added of 1 mL of deuterated buffer (potassium phosphate buffer pH 7.4, 4% NaN_3_, 0.08% TSP in D_2_O) and mixed very well. Six-hundred μL of the supernatant were placed in a 5 mm outer diameter NMR tube. All chemicals were from Sigma (Sigma-Aldrich Corporation, St. Louis, MI, USA).

Liver was excised from each fish and maintained at −80 °C till processing for evaluating caulerpin accumulation levels by ultra-performance liquid chromatography/mass spectrometry (UPLC-MS/MS) as described in a parallel work focusing on biomarker analyses [16].

Not considering the sampling site, the samples were divided in four groups corresponding to fish containing Absent (0 μg/g d.w.), Low (<10 μg/g d.w.), Medium (>10, <50 μg/g d.w.) and High (>50 μg/g d.w.) caulerpin level, respectively.

### 3.2. NMR Measurement

All measurements were performed on a Bruker Avance III NMR spectrometer (Bruker, Karlsruhe, Germany) operating at 400.13 MHz for ^1^H observation, equipped with z axis gradient coil and automatic tuning-matching (ATM). Experiments were run in automation mode after loading individual samples on a Bruker Automatic Sample Changer, interfaced with the software IconNMR (Bruker, Karlsruhe, Germany). A time delay of 5 min was set between sample injection and preacquisition calibrations to ensure complete temperature equilibration (300 K). For each sample, a one-dimensional experiment with a transverse-relaxation-filter incorporating pulse sequence (referred to as Carr-Purcell-Meiboom-Gill spin-echo sequence, CMPG) was run with 128 scans, a total spin-spin relaxation delay of 64 ms, and solvent signal saturation during the relaxation delay. The FIDs were multiplied by an exponential weighting function corresponding to a line broadening of 0.3 Hz before Fourier transformation, phasing, and base line correction. All spectra were referenced to the TSP signal (δ = 0.00 ppm). NMR data were processed using TopSpin 2.1 (Bruker, Karlsruhe, Germany). The metabolites were assigned on the basis of 2D NMR spectra analysis (2D ^1^H *J*res, ^1^H COSY, ^1^H-^13^C HSQC and HMBC) and comparison with published data [17,18,19,20,21,22].

### 3.3. Data Processing

NMR spectra were processed using Topspin 2.1 and visually inspected using Amix 3.9.13 (Bruker, Biospin, Italy). ^1^H NMR CPMG spectra were segmented in rectangular buckets of fixed 0.04 ppm width and integrated. The spectral region between 6.50 and 5.50 ppm and 5.10–4.22 ppm were discarded because of the lack of relevant NMR signals and the residual peak of the suppressed water, respectively. The remaining 185 buckets in the range 9.50–0.50 ppm were then normalized to total area to minimize small differences due to total metabolites concentration and/or acquisition conditions among samples and subsequently mean-centered. The resulting data set was made of the ^1^H NMR spectra bucket values (columns) measured for the above plasma fish samples (rows). The description of statistical analyses refers to Pareto scaling data. The data table generated with all the spectra was submitted to multivariate data analysis.

### 3.4. Multivariate Statistical Analyses

Input variables for statistical analyses were generated via bucketing performed on ^1^H CPMG NMR spectra. Multivariate statistical analysis and graphics were obtained using Simca-P version 14 (Umetrics, Sweden). For multivariate statistical analyses of bucket reduced NMR spectra, different procedures were used: Principal component analysis (PCA), orthogonal partial least square (OPLS) and orthogonal partial least squares discriminant analysis (OPLS-DA). PCA is a way of identifying patterns in data, expressing them to highlight their similarities and/or differences and was used to get an overview of the multivariate profiles. Values falling outside the Hotelling T2 95% confidence limit in score plots are considered outliers. The PCA works by decomposing the X-matrix (buckets linked with the NMR signals) as the product of two smaller matrices (loading and score matrices).

PLS regression is a recent technique that generalizes and combines features from principal component analysis and multiple regressions. Its goal is to predict or analyze a set of dependent variables from a set of independent variables or predictors. This prediction is achieved by extracting from the predictors a set of orthogonal factors called latent variables, which have the best predictive power. PLS regression is particularly useful when we need to predict a set of dependent variables from a (very) large set of independent variables (*i.e.*, predictors). Then, PLS is a projection method that, by using a linear multivariate model, can relate the two blocks of variables, *i.e.*, X and Y. In this work, the PLS method was performed in order to justify the number of *t* latent variables. OPLS, is a modification of PLS, which separates the systematic variation in X into two parts, one that is linearly related to Y and one that is orthogonal to Y. Compared to PLS, the advantage of OPLS is that the model is rotated so that class separation is found in the first predictive component, t1, also referred to as the correlated variation, and variation not related to class separation is seen in orthogonal components, also referredto as the uncorrelated variation, to [1]. This separation of predictive and orthogonal components facilitates model interpretation. In our case X-variables are the value obtained after bucketing the ^1^H NMR spectra and the *Y*-variable is the liver caulerpin content. In this work, OPLS method was performed and chosen for final data analysis and representation due to its advantage for the results interpretation. It was easier to interpret OPLS by partitioning the uncorrelated variations orthogonally from the predictive ones, based on the loading S-plot profile.

The orthogonal partial least squares discriminant technique (OPLS-DA) is the most recently used for the discrimination of samples with different characteristics as it has been shown in several recent studies of metabolomics [45]. OPLS-DA is a modification of the usual PLS-DA method, which filters out variation that is not directly related to the response. Therefore, the further improvements made by the OPLS-DA resides in the ability to separate the portion of the variance useful for predictive purposes from the not predictive variance (which is made orthogonal) overcoming the problems of multicollinearity and autocorrelation of the variables. The robustness and predictive ability of the OPLS and OPLS-DA were described by *R*^2^(cum), *Q*^2^(cum) values and *p*[CV-ANOVA]. *R*^2^ is a cross validation parameter and defined as the proportion of variance in the data explained by the models and indicates goodness of fit; *Q*^2^ is defined as the proportion of variance in the data predictable by the model and indicates predictability, which is extracted according to the internal cross-validation default method of SIMCA-P software; *p*[CV-ANOVA] provides a *p*-value indicating the level of significance of group separation in OPLS analyses [46,47,48,49]. Loading scores describe the correlation between the original variables and the new component variables; VIP parameters essentially measure the degree to which a particular variable explains the Y variance (class membership) and p(corr) represents the loadings scaled as a correlation coefficient (ranging from −1.0 to 1.0) between the model and original data [45]. For each OPLS and OPLS-DA models built, a combination of the loading scores, the variable influence on projection (VIP) parameters and p(corr) were examined, in conjunction with the original spectra, to identify which metabolites contributed most to clustering or a trend observed in the data.

## 4. Conclusions

The present work represents the first study where a metabolomic approach has been used to analyze the blood plasma of fish trophically exposed to an invasive pest. The observed variation of the metabolic profiles of the plasma of *D. sargus* might be the effect either of a single dietary compound contained by *C. cylindracea* or of a combination of bioactive compounds acting in synchrony. While the assessment of the relative contribution of each chemical component of the alga to the observed metabolic alteration needs further interdisciplinary research, the much broader approach that characterizes the present report provides a useful tool both to measure the alterations that are occurring in the fish as a consequence of its alimentary behavior and for better management of marine biological invasion in the Mediterranean Sea. Overall, this study set the stage for the rapid assessment of the metabolic status of *D. sargus* feeding on *C. cylindracea* providing new methodological insights for the understanding of the complex effects affecting natural systems as results of biological invasions.

## Supplementary Files

Supplementary File 1
