# Supplementary material for: 1H NMR Spectroscopy and MVA Analysis of Diplodus sargus Eating the Exotic Pest Caulerpa cylindracea
Source: Mar Drugs. 2015 Jun 5;13(6):3550–66. doi: 10.3390/md13063550 (PMC4483644; doi:10.3390/md13063550)
Supplement: Supplementary File 1 [file marinedrugs-13-03550-s001.pdf]

Supplementary Information

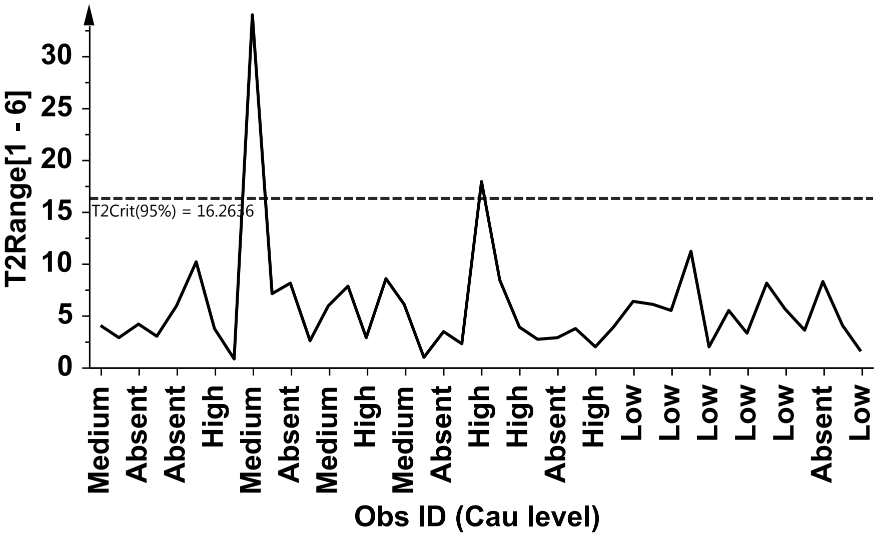

Figure S1. Hotelling's T2 related to PCA analysis on ^{1}H NMR CPMG data for 41 plasma samples.

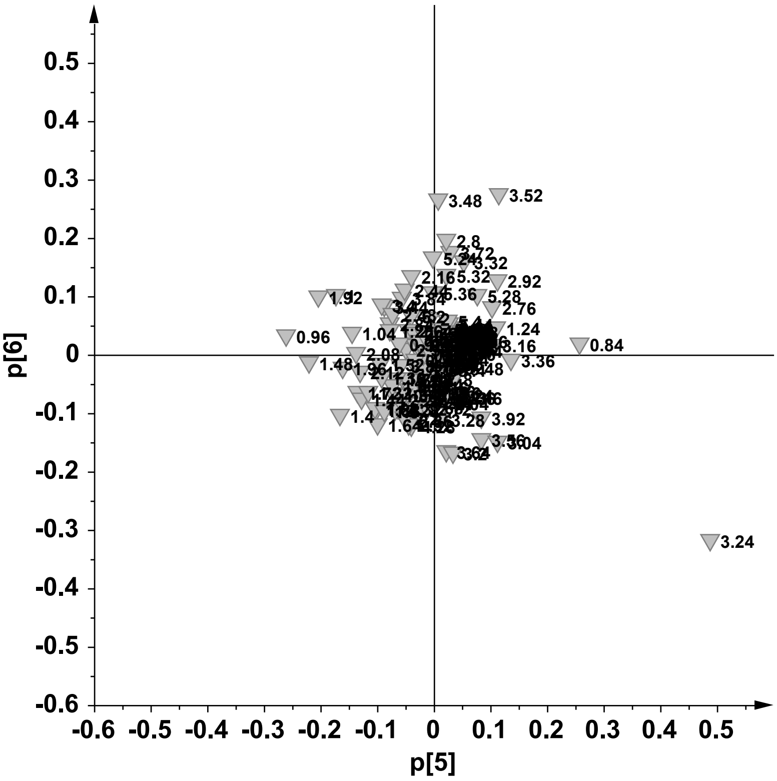

Figure S2. t[5]/t[6] PCA loading plot obtained from ^{1}H NMR CPMG data for 39 plasma samples.

© 2015 by the authors; licensee MDPI, Basel, Switzerland. This article is an open access article distributed under the terms and conditions of the Creative Commons Attribution license (http://creativecommons.org/licenses/by/4.0/).
